# Characterizing nicotine exposure among a community sample of non-daily smokers in the United States

**DOI:** 10.1186/s12889-021-11052-9

**Published:** 2021-05-31

**Authors:** Andréa L. Hobkirk, Vishal Midya, Nicolle M. Krebs, Sophia I. Allen, Lisa Reinhart, Dongxiao Sun, Andrea L. Stennett, Joshua E. Muscat

**Affiliations:** 1 Department of Public Health Sciences, Penn State College of Medicine, Hershey, PA USA; 2 Department of Psychiatry and Behavioral Health, Penn State College of Medicine, Hershey, PA USA; 3 Department of Environmental Medicine and Public Health, Icahn School of Medicine at Mount Sinai, New York City, NY USA; 4 Department of Pharmacology, Penn State College of Medicine, Hershey, PA USA; 5 Bloomberg School of Public Health, Johns Hopkins University, Baltimore, MD USA

**Keywords:** Non-daily smokers, Nicotine exposure, Nicotine dependence, Cotinine, Smoking behavior

## Abstract

**Background:**

Over one-quarter of all smokers in the United States identify as non-daily smokers and this number is projected to rise. Unlike daily smokers who typically maintain consistent levels of nicotine exposure with regular smoking, non-daily smokers have variable patterns of smoking that likely result in high intraindividual variability in nicotine intake. The current study aimed to characterize the weekly intraindividual variability in cotinine and identify smoking-related predictors in nondaily smokers.

**Methods:**

An ecological momentary assessment of 60 non-daily smokers ages 24–57 years was conducted over a consecutive 7-day at-home protocol to log each smoking session, assessments of mood and social activity during smoking, and collection of daily saliva samples in a convenience sample from Pennsylvania, USA. Hierarchical linear regression analyses were conducted to determine the effects of smoking characteristics on total cotinine exposure measured by pharmacokinetic area under the curve and the range, maximum, and minimum cotinine values during the week controlling for demographic variables.

**Results:**

The mean daily cotinine level was 119.2 ng/ml (SD = 168.9) with individual values that ranged from nondetectable to 949.6 ng/ml. Menthol predicted increased total cotinine levels (*P* < 0.05). Shorter time to the first cigarette of the day predicted significantly higher minimum (*P <* 0.05), maximum (*P <* 0.05), and total cotinine values (*P <* 0.05) after controlling for covariates. Negative emotions and social interactions with others were also significantly associated with higher cotinine metrics. There was no significant effect of the nicotine metabolite ratio.

**Conclusions:**

Our findings highlight the variability in nicotine exposure across days among non-daily smokers and point to the role of smoking context in nicotine exposure. The findings suggest the need to develop better assessment methods to determine health and dependence risk and personalized cessation interventions for this heterogeneous and growing group of smokers.

## Background

In the United States, the prevalence of cigarette smoking decreased from 21% in 2005 to 14% in 2017 [1]. Accordingly, the number of non-daily smokers is expected to rise as tobacco use becomes more expensive and restricted in the U. S [2, 3]. Approximately one-quarter to one-third of all smokers in the U.S. identify as non-daily smokers who do not smoke a cigarette every day [2, 4]. While non-daily smoking is linked to fewer health risks than daily smoking, non-daily smokers still have substantially higher rates of lung cancer and cardiovascular disease than the general population [5]. National trends suggest that low frequency smokers are becoming more nicotine dependent than in the past, possibly because this group comprises former heavy smokers [6]. A large national survey found that non-daily smokers were more likely to make a quit attempt and maintain abstinence at 90 days than daily smokers [7]. However, the rates of abstinence for non-daily smokers with and without a prior history of daily smoking are still quite low, 27 and 18% respectively, especially considering that approximately half of non-daily smokers are not nicotine dependent [7, 8]. Thus, non-daily use still puts smokers at risk for adverse health outcomes.

Nicotine is the primary psychoactive component in cigarettes and the main driver of cigarette dependence [9]. Although nicotine itself is not carcinogenic and makes only minor contributions to the development of cardiovascular disease, nicotine exposure can serve as a biomarker of negative health outcomes since it increases in parallel with other toxins and chemicals in cigarette smoke [10–13]. Nicotine, and its metabolite cotinine, can be measured in blood, saliva, and urine, making it an accessible and reliable predictor of disease risk and dependence [14]. Despite the rising prevalence of non-daily smoking in the U.S., we still know little about the levels, consistency, and correlates of nicotine exposure for non-daily smokers. One study sampled urinary cotinine at a single time point and found levels to be nearly three times lower among non-daily (aka., intermittent) compared to daily smokers [15]. The amount of exposure per cigarette was similar for daily and non-daily smokers in this study [15].

Not surprisingly, non-daily smokers have lower levels of nicotine dependence than daily smokers [16]. This finding has been replicated across different facets of dependence, including behavioral measures of dependence such as smoking motives, and self-perceived loss of autonomy over smoking [8, 16]. Given the lower levels of nicotine exposure, smoking for non-daily smokers may be even more dependent on environmental and internal stimuli such as mood, activity, and social setting than for daily smokers [17]. Our previous report using ecological momentary assessments among non-daily smokers found that smoking was most likely to occur during positive moods and leisure activities compared to negative and neutral mood states or performative and social activities [18]. Smoke intake, however, is not the only determinant of nicotine exposure for smokers. For example, compared to white smokers, black smokers have higher nicotine metabolite levels per cigarette [19]. This racial difference becomes even more pronounced with fewer cigarettes smoked per day [20]. A similar effect has also been found for menthol compared to regular tobacco smokers [20]. Even mood state, such as positive affect, has been linked to increased nicotine boosts during cigarette smoking [21]. We still have a limited understanding of what factors influence nicotine exposure for non-daily smokers, and if nicotine exposure is moderated by demographic and smoking factors, like race and flavor preference, as it is for daily smokers.

Unlike daily smokers that typically maintain consistent levels of blood nicotine to avoid withdrawal symptoms, non-daily smokers report a wide range in the number of cigarettes they smoke per day and the number of days of abstinence between smoking days [8, 9]. Thus, non-daily smokers are a heterogeneous group that may have high variability in their day-to-day nicotine exposure [8, 22]. In other words, the cotinine value on one day may be considerably different to the value on another day. Therefore, it is important to study intraindividual differences in nicotine exposure, including total exposure and consistency in exposure across time, to obtain an accurate picture of nicotine exposure for non-daily smokers.

Our primary aims for the current study were to: 1) characterize the intraindividual variability in nicotine exposure for non-daily smokers, and 2) identify demographic and smoking factors associated with nicotine exposure for non-daily smokers. To accomplish these aims, we measured salivary cotinine from non-daily smokers over a 7 day collection period. While mean level of daily nicotine exposure is a meaningful metric for understanding true nicotine exposure for daily smokers, non-daily smokers, on the other hand, likely have more variability in day-to-day nicotine exposure. A one time single day measure of cotinine not only fails to capture the day-to-day variability, but may also provide an inaccurate view of the true nicotine exposure by not accounting for days when no cigarettes were smoked. To overcome this challenge, we used four different metrics to characterize intraindividual differences in nicotine exposure, including area under the curve (AUC) to capture total weekly exposure, maximum and minimum daily values to capture level of exposure on any 1 day, and the range of daily values to measure consistency of exposure across days. We then identified demographic, smoking, and contextual-related correlates of nicotine exposure to explore potential predictors and outcomes of tobacco exposure for non-daily smokers.

## Methods

### Participant recruitment

Non-daily smokers (*n* = 60) in the United States were recruited as a sub-sample of the Pennsylvania Adult Smoking Study (PASS) [23]. The PASS study investigated whether socioeconomic status predicts smoke exposure in daily smokers [24], and the current sub-study was designed to observe factors related to smoking for non-daily smokers specifically. Online advertisements, flyers, and peer-referrals were used for recruitment from 2014 to 2015. Participants were eligible if they reported smoking 4–24 days out of the past 30, and had this pattern for at least 6 months. Interested individuals were excluded if they reported that > 50% of their tobacco use was a product other than a cigarette, were currently pregnant, or engaged in active cessation attempts.

### Procedures

If eligible after phone screening, participants were invited to an initial in-person visit where they provided written informed consent and completed a battery of questionnaires that included measures of nicotine dependence. During a 7-day at-home protocol, participants completed daily smoking logs, measures of smoking topography, ecological momentary assessments (EMA) during each smoking session (described below), and collected one saliva sample at approximately the same time each day for nicotine metabolite analysis. Participants returned all logs, topography equipment, and saliva samples at a final visit following the 7-day collection period. Topography data is not presented in this manuscript and a thorough description of the emotions and activities reported using EMA during each cigarette session has been published [18]. REDCap (Research Electronic Data Capture) tools hosted at the Penn State Milton S. Hershey Medical Center and College of Medicine were used for all data collection and management [25]. All study procedures were approved by the Institutional Review Board at the Penn State College of Medicine (037860EP).

### Salivary cotinine

Participants provided daily saliva samples for nicotine metabolite analysis. Cotinine has a longer half-life compared to nicotine, approximately 16 h for cotinine versus 2 h for nicotine, making it a sensitive measure of smoking even among light and non-daily smokers [26, 27]. For daily saliva collection, participants placed a SalivaBio Oral Swab (Salimetrics, State College, Pennsylvania) under their tongues for 2 min. Participants stored the swabs in their home freezer until the final data collection visit. Swabs were kept frozen (− 80 °C) in the laboratory until High Sensitivity Salivary Cotinine Quantitative Enzyme Immunoassay (ELISA; Salimetrics, State College, Pennsylvania) was carried out as directed by the manufacturer for the total sample. For 17 participants, immunoassay results were compared to assays conducted using mass spectrometry methods previously described [28] and modified for increased dilution in saliva samples using Triple TOF 5600 (AB SCIEX, Concord, Ontario, Canada). The same mass spectrometry methods were used to calculate cotinine and trans-3′-hydroxycotinine (3HC) on each participant’s initial saliva sample to determine nicotine metabolite ratio (NMR), calculated as 3HC divided by cotinine. Continuous NMR values were log transformed for analysis. Similar to previous studies, participants in the lowest quartile of NMR values (≤0.31) were categorized as slow metabolizers and those in the top three quartiles as normal metabolizers [29]. To determine the reliability of cotinine over the course of a single day for non-daily smokers, six participants provided four extra saliva samples (5 total) three to four hours apart on 1 day. These were analyzed using immunoassay.

### Baseline assessment

Participants provided basic demographic information and detailed smoking histories. Those who answered “yes” to the question “Have you ever smoked daily for 6 months or longer?” were categorized as a “converted” non-daily smoker (i.e., converted to non-daily smoking from daily smoking) and those who answered no were categorized as a “native” non-daily smoker, as has been done in prior research [8].

The Hooked on Nicotine Checklist (HONC) measured loss of perceived autonomy over smoking using 10-items that assess past recall of withdrawal, craving, and quit attempts (e.g., Do you ever have strong cravings to smoke?) [30]. HONC scores range from 0 (low) to 10 (high) and internal consistency was good in the current sample as expected (Cronbach’s *α* = 0.70). The HONC is more sensitive to dependence among very light smokers, likely because the score is not weighted heavily by the number of cigarettes smoked per day like other dependence measures [31]. While the HONC was developed for a dichotomous scoring system with any item endorsement indicating loss of autonomy over smoking [32], we measured it as a continuous marker of the degree of autonomy as has been done previously with non-daily smokers [16, 31].

### Event-level assessment

At each ad-lib smoking session, participants answered a series of questions about their current emotion, activity, time of day, urge, if they were alone, and if they were currently consuming alcohol. Activities were coded into four categories: leisure (e.g., watching TV, at home, on the computer, and drinking coffee), performative (e.g., completing chores, driving, getting child ready for school), social (e.g., at a party, at a friend’s home, at the casino), and interactive (e.g., talking on the phone, arguing with another, business calls, and having a conversation). Emotions were categorized as: positive, negative, neutral, and mixed. The details of these categories, reliability of the coding procedure, and results of the EMA analysis are described in a prior publication [18].

### Data analysis

#### Patterns of daily cotinine

**Fig. 1:**
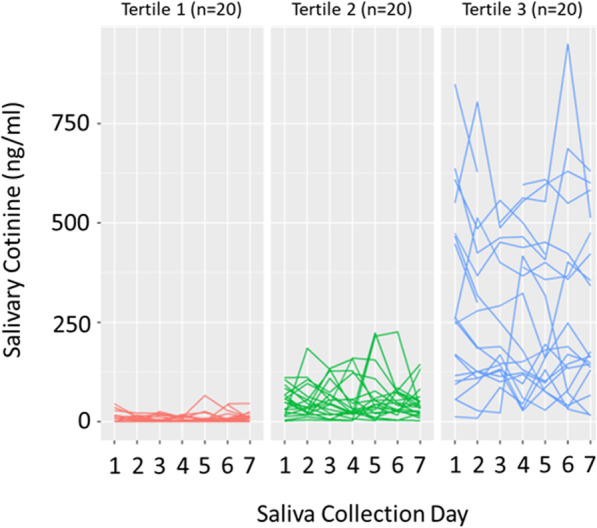
Display of the intraindividual variability of daily salivary cotinine values for each participant in the sample. For display purposes, the sample was grouped into tertiles based on each participant’s mean cotinine value. Each line represents the daily salivary cotinine (ng/ml) values for a single participant

Data were analyzed using R statistical software (version 3.6.1 (2019-07-05)) and SPSS (version 26). We calculated four descriptive metrics of daily cotinine including the area under the curve (AUC), maximum exposure, minimum exposure, and range of daily values. The AUC is a pharmacokinetic measure that represents total drug exposure over time. AUC for total nicotine exposure during the week was calculated using the linear trapezoidal method. The cotinine range provided a measure of consistency in cotinine values during the week and was calculated as the minimum value subtracted from the maximum value. We divided the sample into tertiles based on the mean cotinine value and plotted each participant’s seven daily cotinine values to show graphically the variability in cotinine levels across days and participants (see Fig. 1).

#### Associations of cotinine metrics with smoking characteristics, dependence, and activities and emotions

Cotinine metrics (AUC, min, max, range) were regressed onto predictor variables including demographics, smoking characteristics, activities, and emotions in separate hierarchical linear regression Covariates included age, sex, race, total family annual income, and marital status. Significance level was set at *p*-value <.05, and we also applied a Bonferroni correction under the assumption of multiple hypothesis testing where significance level was set at *p*-value <.003.

## Results

### Sample characteristics and tobacco use patterns

The sample included 60 non-daily smokers ages 24 to 57 years, who were predominantly white (78%) and non-Hispanic (90%) with an average total family income of $49,414 and more than a high school degree (82%). Approximately half of the total sample was female (52%) and married or living with a partner (45%).

Participants reported smoking a mean of 3.2 (SD = 1.8) cigarettes per day and 13.9 (SD = 5.3) days out of the past 30. The mean age of smoking onset was 19.5 (SD = 5.3) years, and the mean duration of nondaily smoking was 8.1 (SD = 7.4) years. Sixty percent of the sample converted from daily smoking to non-daily smoking. The converted non-daily smokers reported switching from daily smoking 6.7 (SD = 7.1) years ago on average, and previously smoking daily for a mean of 8.8 (SD = 7.4) years. Thirty-two participants (53%) endorsed use of menthol cigarettes and 62% reported a lifetime quit attempt. The sample reported smoking an average of 424.3 min after waking (SD = 286.7). The mean HONC dependence score was 4.1 (SD = 2.1).

### Cotinine metrics

**Fig. 2:**
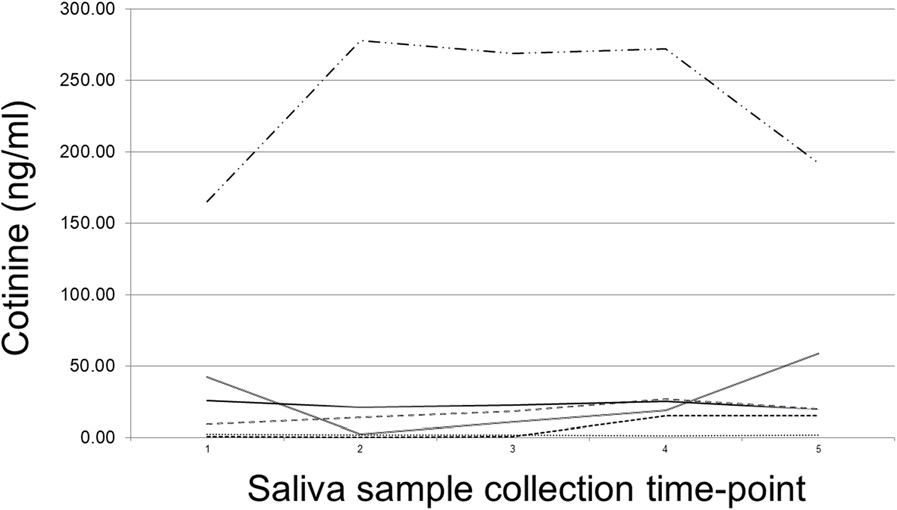
Five cotinine values derived from saliva samples collected every 3–4 h during a single day for six randomly selected participants

Participants were grouped into tertiles based on their mean cotinine values to illustrate the variability of cotinine within and between participants (Fig. 1). The sample had a mean daily cotinine value of 119.2 ng/ml (SD = 168.9) with daily values that ranged from nondetectable to 949.6 ng/ml. The mean range was 113.6 (SD = 110.5). The mean AUC was 690.8 (SD = 969.7). The mean nicotine metabolite ratio (3HC/cotinine) was 0.6 (SD = 0.4), and 25% (*n* = 15) of the sample was categorized as a slow metabolizer with a ratio ≤ 0.3. Figure 2 displays single-day repeated cotinine values (ng/ml) for the six participants randomly selected to collect five repeated cotinine samples over the course of a single day. Overall, the single-day cotinine values remained stable, especially when mean values were less than 50 ng/ml.

### Linear hierarchical regression analyses

#### Biological and behavioral smoking characteristics

Controlling for demographic variables, shorter time to the first cigarette of the day was associated with significantly higher min, max, and AUC cotinine values (Table 1). Preference for menthol cigarettes was associated with significantly higher min, max and AUC cotinine values, but only higher min met significance with the Bonferroni adjustment. No other smoking variables including NMR were significant predictors of cotinine measures.

**Table 1:** Results of hierarchical linear regression analyses of cotinine measures regressed onto smoking factors

Cotinine outcome measure	Maximum value	Minimum value	Range of values	Total AUC
	B (SE)	B (SE)	B (SE)	B (SE)
Characteristics, Adjusted *R*^2^	0.2	0.3	0.1	0.3
Age smoking fairly regularly (years)	1.8 (6.4)	−2.0 (3.7)	3.8 (3.3)	1.5 (26.0)
Duration of non-daily smoking (months)	1.2 (5.4)	0.9 (3.2)	0.3 (2.8)	3.8 (22.0)
Converted vs. native non-daily smoker	−29.3 (72.5)	−19.2 (42.6)	−10.2 (37.7)	− 46.1 (297.0)
Menthol vs. regular preference	142.7 (62.8)^b^	118.3 (36.9)^a^	24.4 (32.6)	727.9 (257.2)^b^
NMR (log)^d^	132.8 (88.2)	60.6 (51.8)	72.1 (45.8)	471.2 (361.2)
Time to first cigarette after waking (minutes)	−0.4 (0.1)^a^	−0.3 (0.1)^a^	− 0.2 (0.1)^b^	−1.9 (0.5)^a^
Nicotine dependence severity^c^	−0.6 (17.0)	− 0.9 (10.0)	0.3 (8.8)	−5.4 (69.7)
Activities, Adjusted *R*^2^	0.3	0.2	0.3	0.3
Performative	14.2 (10.0)	6.0 (6.5)	8.2 (4.7)	49.5 (42.7)
Leisure	9.9 (6.7)	4.2 (4.4)	5.7 (3.2)	44.7 (28.5)
Social	2.2 (8.5)	−5.5 (5.6)	7.6 (4.0)	−15.5 (36.2)
Interactive	72.0 (19.3)^a^	40.2 (12.7)^a^	31.8 (9.1)^a^	310.6 (82.6)^a^
Emotions, Adjusted *R*^2^	0.3	0.2	0.4	0.2
Positive	13.7 (7.4)	−0.34 (4.9)	14.0 (3.2)^a^	34.1 (32.6)
Negative	33.1 (8.7)^a^	17.6 (5.7)^a^	15.5 (3.8)^a^	126.5 (38.3)^a^
Mixed	53.5 (29.4)	38.1 (19.4)	15.4 (12.9)	256.0 (130.2)
Neutral	−3.4 (12.2)	1.1 (8.0)	−4.4 (5.4)	14.8 (54.0)

Covariates included years of age, sex, race, total family annual income, and marital status

*AUC* area under the curve, *NMR* nicotine metabolite ratio

^a^*p* < .003, ^b^ *p* < .05

^c^Hooked on Nicotine Checklist total score

^d^Results did not vary when continuous NMR was replaced by slow vs. normal metabolizer NMR variable

#### Smoking activities and emotions

In total, the sample reported 561 smoking experiences and their associated emotions and activities. On the smoking logs, participants reported an average of 3.8 (SD = 1.8) days of smoking during the collection week and 2.4 (SD = 1.4) cigarettes per smoking day. Three participants reported one instance of using a tobacco product other than a cigarette (i.e., electronic cigarette, snuff, and hookah), one participant reported two instances of cigar use, and one participant reported 19 instances of electronic cigarette use during the collection week. All results remained the same with the latter participant excluded from analysis. Participants reported being alone for 58.9% and using alcohol for 23.5% of all reported experiences of tobacco use. The sample reported engaging in leisure activities during smoking most often (*n* = 272, 48.5%) followed by performative (*n* = 161, 28.7%), social (*n* = 90, 16.0%), and interactive (*n* = 38, 6.8%) activities. The sample reported experiencing positive emotions during smoking most often (*n* = 262, 46.7%), followed by negative (*n* = 179, 31.9%), neutral (*n* = 91, 16.2%), and mixed (*n* = 29, 5.2%) emotions. Controlling for demographic variables, more interactive activities and negative emotions were associated with significantly higher min, max, range and AUC cotinine measures (Table 1). More positive emotions was significantly associated with a higher range in cotinine values.

## Discussion

The current study measured nicotine exposure over a 7-day collection period among a sample of non-daily smokers. There was considerable variation in cotinine levels across participants and between days with some subjects showing consistent low levels day to day and others showing higher and more variable patterns. Intraday variation was low among the five randomly selected participants who took multiple measurements in a single day. The variability in nicotine exposure in our sample map onto a prior study that conducted a latent class analysis among college students’ smoking behavior [33]. This study found three classes of non-daily smokers, including a group who smoked several cigarettes a day on more than half the days in a month, a group who smoked several cigarettes a day primarily in social situations on the weekends, and a group who only occasionally smoked all or part of a cigarette [33]. This study did not measure nicotine exposure, but one could expect that cotinine values for these three groups would look similar to our tertiles, with relatively high, but variable levels in the first group, moderate cotinine levels on few days of the week in the second group, and consistently low levels in the last group.

Since nicotine levels correlate with other biomarkers of tobacco-related carcinogens and toxins in daily smokers [14], non-daily smokers might also experience varying levels of harmful exposure. Some non-daily smokers had high levels of cotinine that were similar to that in daily smokers, while others maintained very low levels throughout the entire week. Those participants who reached high cotinine values on a single day tended to have more variability in their levels throughout the week. A review of the health effects of smokers who smoke just a few cigarettes per day indicated that harm occurs even at low levels of smoking and it can be inferred that nondaily smokers also are at increased risk [5].

Cotinine measures were not associated with loss of autonomy. The HONC scores in the current study were similar to that reported for other non-daily smokers [16]. In addition, nicotine exposure was not higher for converted compared to native daily smokers, which have also been shown to have higher rates of nicotine dependence [6]. All cotinine metrics were associated with time to first cigarette after waking. Shorter time to first cigarette after waking is one of the strongest predictors of higher nicotine dependence severity among daily smokers and has previously been associated with cotinine levels for daily smokers [34, 35]. The findings confirm that non-daily smokers are a heterogenous group with varying levels of nicotine dependence.

In our sample, menthol preference predicted higher minimum cotinine values throughout the week. Menthol smoking was also related to higher AUC and maximum cotinine values, although these predictors did not reach statistical significance with Bonferroni correction. The U.S. Food and Drug Administration (FDA) concluded that the use of menthol cigarettes is more common among low income smokers and related to higher levels of dependence [36]. Although there are some reports that menthol is associated with higher nicotine intake in daily smokers, an FDA review did not confirm this finding [36, 37]. Among our sample, menthol smoking was not associated with higher scores on the HONC in a post-hoc analysis (*p* = 0.30).

Our EMA results from the current study and a prior analysis of the sample found that smoking occurred most frequently during positive moods and activities of leisure [18]. Non-daily smokers have stronger stimulus control over their smoking habits than daily smokers [17]. Stimulus control occurs when a smokers’ mood, activity, and environmental context become important predictors of smoking behavior, rather than nicotine withdrawal alone [17]. This could explain why our participants with low levels of nicotine exposure still reported perceived symptoms of nicotine dependence (e.g., mean HONC = 4.1). We found that these contextual factors were also important predictors of cotinine in our sample. Smoking during interactive activities and negative emotional states was related to higher min, max, and AUC cotinine values. Positive emotions were linked with more variability in nicotine exposure across days. This could have several explanations. Given the relatively higher levels of dependence among this group, these smokers may experience withdrawal symptoms that induce negative emotions, like irritability and anxiety, which precede and then accompany their next smoking experience. Another potential explanation is that our participants with high cotinine levels may smoke to alleviate perceived stress. Experimentally induced stress has been shown to trigger negative affect and craving among heavy and light smokers and is commonly reported as a barrier to cessation [38, 39]. Future research exploring real-time smoking motives among non-daily smokers may help to elucidate why negative emotions are associated with higher cotinine levels for this group and how we might intervene on these emotions to reduce smoking behavior (see [40] for review).

The current study had several limitations. The sample, comprised primarily of non-Hispanic White smokers, was not nationally representative. For this reason our results may not generalize to Black and Hispanic smokers who are known to have higher rates of non-daily smoking than the general population [41]. In addition, our EMA relied on participants’ initiative to record each smoking session and some participants may have under-reported the number of cigarettes smoked; however, the sample’s self-reported CPD was consistent with prior studies of adults and adolescents [15]. Finally, our study was underpowered to detect small effects and may have missed meaningful associations. For example, lower total family income was associated with higher cotinine exposure, but was not significant. Future research with larger, representative samples will help clarify the relevance of these associations with nicotine exposure for non-daily smokers.

## Conclusions

The current study highlights that nicotine exposure as measured by cotinine among non-daily smokers is as variable as their self-reported patterns of smoking behavior. Nicotine exposure for non-daily smokers varies with behavioral measures of dependence (i.e., time to first cigarette) and contextual factors that have also been found with samples of daily smokers. With a wide range in nicotine exposure and consistently low nicotine dependence and cessation rates, some non-daily smokers may not fit with the long-held theory that nicotine is the primary factor that sustains smoking addiction [42]. Instead, these findings point to the need for more detailed assessments of smoking behavior to guide the development of effective cessation interventions for this variable group of smokers. Future research could inform our limited understanding of the biobehavioral mechanisms that contribute to individual differences in nicotine exposure among non-daily smokers and inform the development of personalized interventions that help non-daily smokers manage internal and external cues for smoking.

## Data Availability

The de-identifiable data that support the findings of this study are available from the corresponding author, upon reasonable request and transfer through an institutional data sharing agreement.

## Abbreviations

AUC: Area Under the Curve
PASS: Pennsylvania Adult Smoking Study
EMA: Ecological Momentary Assessments
REDCap: Research Electronic Data Capture
3HC: Trans-3′-hydroxycotinine
NMR: Nicotine Metabolite Ratio
HONC: Hooked on Nicotine Checklist
